# From Ecological Stoichiometry to Biochemical Composition: Variation in N and P Supply Alters Key Biosynthetic Rates in Marine Phytoplankton

**DOI:** 10.3389/fmicb.2017.01299

**Published:** 2017-07-12

**Authors:** Julia Grosse, Amanda Burson, Maayke Stomp, Jef Huisman, Henricus T. S. Boschker

**Affiliations:** ^1^Department of Marine Microbiology and Biogeochemistry, Royal Netherlands Institute for Sea Research, and Utrecht University Den Burg, Netherlands; ^2^Department of Aquatic Microbiology, Institute for Biodiversity and Ecosystem Dynamics, University of Amsterdam Amsterdam, Netherlands

**Keywords:** marine phytoplankton, compound specific isotope analysis, nutrient competition, N:P ratios, ^13^C-labeling, ecological stoichiometry

## Abstract

One of the major challenges in ecological stoichiometry is to establish how environmental changes in resource availability may affect both the biochemical composition of organisms and the species composition of communities. This is a pressing issue in many coastal waters, where anthropogenic activities have caused large changes in riverine nutrient inputs. Here we investigate variation in the biochemical composition and synthesis of amino acids, fatty acids (FA), and carbohydrates in mixed phytoplankton communities sampled from the North Sea. The communities were cultured in chemostats supplied with different concentrations of dissolved inorganic nitrogen (DIN) and phosphorus (DIP) to establish four different types of resource limitations. Diatoms dominated under N-limited, N+P limited and P-limited conditions. Cyanobacteria became dominant in one of the N-limited chemostats and green algae dominated in the one P-limited chemostat and under light-limited conditions. Changes in nutrient availability directly affected amino acid content, which was lowest under N and N+P limitation, higher under P-limitation and highest when light was the limiting factor. Storage carbohydrate content showed the opposite trend and storage FA content seemed to be co-dependent on community composition. The synthesis of essential amino acids was affected under N and N+P limitation, as the transformation from non-essential to essential amino acids decreased at DIN:DIP ≤ 6. The simple community structure and clearly identifiable nutrient limitations confirm and clarify previous field findings in the North Sea. Our results show that different phytoplankton groups are capable of adapting their key biosynthetic rates and hence their biochemical composition to different degrees when experiencing shifts in nutrient availability. This will have implications for phytoplankton growth, community structure, and the nutritional quality of phytoplankton as food for higher trophic levels.

## Introduction

Changes in nutrient availability affect the C:N:P ratio of primary producers, both through physiological acclimation and shifts in species composition. In turn, these shifts in the elemental composition of primary producers can have major implications for nutrient cycling and their quality as food for herbivores, which are key focal research areas of the rapidly expanding field of ecological stoichiometry (Sterner and Elser, 2002; Hessen et al., 2004; Vrede et al., 2004; Persson et al., 2010). However, although C:N:P ratios are easily measured, an often voiced criticism is that they do not provide detailed information on changes in the biochemical composition of primary producers in terms of, e.g., amino acids (AA), fatty acids (FA) and carbohydrates (CH), DNA and RNA (Anderson et al., 2004; Raubenheimer et al., 2009). The biochemical composition of primary producers is important for their own growth and survival, and plays a key role in many plant-herbivore interactions. For instance, most herbivores cannot synthesize all AA and FA themselves, but rely on the provision of essential AA and FA from the primary producers in their diet (Müller-Navarra, 1995; Fink et al., 2011). Therefore, a deeper understanding of how changes in environmental nutrient availability affect the biochemical composition of primary producers would be a major next step.

Many coastal waters have witnessed major changes in nutrient input during the past several decades. The North Sea provides a good example. Between the early 1960s and mid-1980s mean annual concentration of dissolved inorganic N tripled, while at the same time P concentrations doubled, resulting in coastal eutrophication (Hickel et al., 1993). Effects of eutrophication included an increase in phytoplankton biomass (Cadée and Hegeman, 2002), shifts in species composition (Philippart et al., 2000), the formation of toxic algal blooms (Riegman et al., 1992; Lancelot et al., 2007), changed trophic food web structures (Van Beusekom and Diel-Christiansen, 2009) and the development of hypoxia (Westernhagen and Dethlefsen, 1983). In response, members of the OSPAR Convention (Oslo/Paris Convention for the Protection of the Marine Environment of the North-East Atlantic) agreed to lower riverine N and P inputs to the North Sea by at least 50% compared to the year 1985 (OSPAR, 1988). Nutrient reduction efforts resulted in an effective P removal from domestic and industrial wastewater. By 2002, many countries reached and even exceeded the goal for P, by decreasing P inputs by 50–70%. However, decreasing N inputs was less successful and N loads were only lowered by 20–30% (Lenhart et al., 2010; OSPAR, 2010; Passy et al., 2013). As a consequence, riverine N:P inputs to the coastal North Sea currently greatly exceed the Redfield ratio of 16:1 (Radach and Pätsch, 2007; Thieu et al., 2010; Grizzetti et al., 2012; Burson et al., 2016).

Similar patterns have been observed in other coastal waters. Effective P removal in combination with a global increase in the application of N fertilizers has increased the N:P ratios of many riverine nutrient inputs to coastal waters (Turner et al., 2003; Grizzetti et al., 2012; Glibert et al., 2014). Consequently, P limitation is currently becoming more prevalent in river-influenced coastal seas, not only in the North Sea (Burson et al., 2016) but also in, e.g., the Gulf of Mexico and the South China Sea (Sylvan et al., 2007; Xu et al., 2008), challenging the classical view that N is the main limiting nutrient in marine coastal systems (Hecky and Kilham, 1988; Howarth and Marino, 2006). In the North Sea, this pattern is further confirmed by high nearshore POC:POP ratios (400–700) during the phytoplankton spring bloom, indicative of severe P deficiency of coastal phytoplankton (Burson et al., 2016). Lab studies have shown that P-deficient phytoplankton may cause lower growth rates in marine zooplankton (Malzahn et al., 2007; Malzahn and Boersma, 2012; Schoo et al., 2013), and that the elevated C:P ratios of this zooplankton can, in turn, have detrimental effects on larval growth of economically valuable species such as herring (Malzahn et al., 2007) and European lobster (Schoo et al., 2014). So far, however, little is known about the implications of these changes in nutrient limitation for the biochemical composition of marine phytoplankton.

In recent years, advances in compound-specific isotope analysis by either gas chromatography (GC) or liquid chromatography (LC) in combination with isotopic ratio mass spectrometry (IRMS) have made it possible to obtain specific isotope information from a wide range of biomolecules in complex mixtures (e.g., McCullagh et al., 2006; Boschker et al., 2008; Veuger et al., 2012). Now, ^13^C stable isotopes can be used in the same way as in primary production measurements but on a more detailed compound-specific level, by measuring the incorporation of photosynthetically fixed carbon into individual FA, AA, and CH (Grosse et al., 2015, 2017). This opens up opportunities to study the biochemical composition and nutritional quality of phytoplankton in much further detail.

In this study, we explore how changes in N and P loads may potentially affect the biochemical composition of coastal marine phytoplankton. As model system, we inoculated laboratory chemostats with mixed phytoplankton communities sampled from the North Sea. This experimental approach enabled a systematic investigation of the effects of different N:P supply ratios on resource limitation, biochemical composition and biosynthesis rates of the phytoplankton community using compound-specific isotope analysis.

## Materials and Methods

### Collection of Inoculum

Samples for field inoculum were taken from eight stations along a 450 km long transect from the Dutch coast towards the center of the North Sea between 15 and 22 March 2013 onboard the Dutch research vessel RV Pelagia (Grosse et al., 2017). At each station a 20 L carboy was rinsed and filled with water collected at 7 m depth. Water was passed through a 200 μm mesh then bubbled for 30 min each with CO_2_ and N_2_ gas to eliminate grazers. The carboys were kept at 4°C until initiation of chemostat experiments at the University of Amsterdam. Equal portions of water from each station were combined, resulting in a single inoculum for the chemostat experiments containing a mix of phytoplankton from all eight stations along the entire 450 km transect (for additional details, see Burson et al. (2016).

### Chemostat Set-Up

**FIGURE 1:**
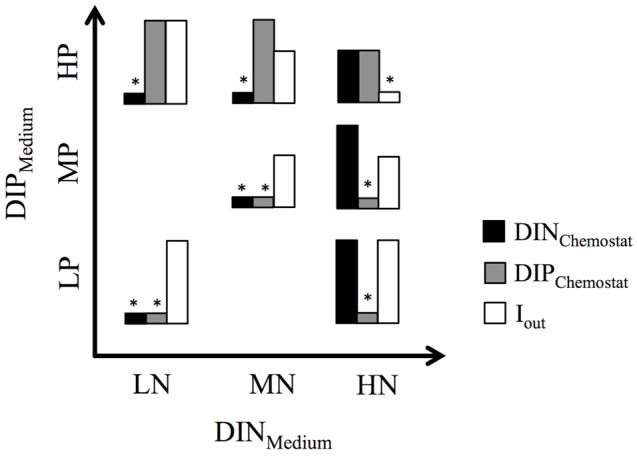
Bar graphs show the relative distribution of nutrient concentrations (DIN_Chemostat_, DIP_Chemostat_) and light penetration (I_out_) in the seven chemostat experiments. ^∗^above bars indicate the resulting resource limitation. Experimental set-up is based on three levels of dissolved inorganic nitrogen (DIN_Medium_) and phosphorus (DIP_Medium_) in the mineral medium supplied to the chemostats.

Within 2 days after the cruise ended, seven flat-walled chemostats (mixing depth: 5 cm) were set up according to Huisman et al. (2002), using full-spectra white fluorescent bulbs as light sources and magnetic stir bars to minimize accumulation of sticky and heavy species. The incident light intensity at the front surface of each chemostat was set at 40 μmol photons m^-2^ s^-1^ and the dilution rate at 0.2 d^-1^. Irradiance passing through the chemostat vessel (I_out_) was measured with a light meter (LI-250 LI-COR, NE, United States) at ten regularly spaced positions at the back surface of the chemostat. The seawater inoculum was added to fill half the chemostat’s volume (0.5 L) and was topped off with one of seven artificial seawater media using peristaltic pumps. Media exhibited different combinations of dissolved inorganic nitrogen (DIN_Medium_) and phosphate (DIP_Medium_) at low (LN/LP), medium (MN/MP), or high (HN/HP) concentrations, hence also differed in their DIN:DIP_Medium_ ratios (**Table 1** and **Figure 1**). Inorganic carbon was added in two ways; as sodium bicarbonate to media (0.5 mM final concentration) and as CO_2_ in filtered air, which was bubbled through the chemostats. The chemostats were run as a competition experiment until phytoplankton communities established steady state conditions (91 days) and were then harvested for the carbon fixation experiment.

**Table 1:** Concentrations and ratios of nutrients in the different media and in chemostats when phytoplankton communities reached steady state conditions.

	MNHP	LNHP	MNMP	LNLP	HNHP	HNMP	HNLP
**Nutrients**					
DIN:DIP_Medium_	1.28	0.512	16	16	16	200	500
DIN_Medium_ (μM)	160	64	160	64	2000	2000	2000
DIP_Medium_ (μM)	125	125	10	4	125	10	4
DIN:DIP_Chemostat_	0.04	0.04	1	2	6	275	2380
DIN_Chemostat_ (μM)	2	2	3	4	181	825	1190
DIP_Chemostat_ (μM)	49	46	3	2	29	3	0.5
**Community Composition**					
Diatoms (%)	23	**81**	**84**	**86**	38	6	**86**
Green algae (%)	15	4	5	1	**57**	**87**	9
Cyanobacteria (%)	**62**	15	11	13	5	6	5
**Light penetration** I_out_ (μmol photons m^-2^ s^-1^)	17	24	19	26	0.4	9.5	23
**Biochemical parameters**					
POC (mM)	8.21	3.46	9.63	3.04	26.31	13.35	3.81
PON (mM)	0.334	0.180	0.365	0.127	3.152	1.144	0.485
POP (mM)	0.011	0.010	0.007	0.004	0.060	0.010	0.003
POC:PON	25	19	26	24	8	12	8
POC:POP	746	346	1376	760	439	1335	1270
PON:POP	30	18	52	32	53	114	162
C-fixation (nmol C μmol POC^-1^ d^-1^)	44	73	52	56	83	109	191
DIN requirement (nmol DIN μmol POC^-1^ d^-1^)	1.8	3.8	2.0	2.3	9.9	9.3	24.3
DIP requirement (nmol DIP μmol POC^-1^ d^-1^)	0.06	0.22	0.04	0.07	0.19	0.08	0.14

*Chemostats are sorted left to right by increasing DIN:DIP_*Chemostat*_ ratios. Contribution of the different phytoplankton groups to total biovolume is also shown, with the highest contributing group in bold. Light penetration indicates light availability and particulate organic carbon (POC), nitrogen (PON), and phosphorus (POP) and their ratios are shown. DIN and DIP requirements were calculated using C-fixation rates, POC:PON and POC:POP ratios.*

### Carbon Fixation Experiment

Chemostats were harvested by transferring 1 L of culture into 1.2 L culture flasks. From there, initial and unlabeled subsamples were taken for dissolved inorganic carbon (DIC), nutrient concentrations, particulate organic C, N, and P (POC, PON, POP), and biomolecules (AA, FA, CH). Nutrient samples were filtered through a 0.2 μm Acrodisc filter and stored at 4°C until analysis. DIC samples were also filtered through a 0.2 μm Acrodisc filter, sealed bubble-free in a 10 mL crimp vial and stored at 4°C until analysis. Samples for POC/PON, POP and biomolecules were taken by filtering 30–100 mL per analysis (depending on biomass) over pre-combusted GF/F filters (Whatman, 4 h at 450°C). POC/PON and POP filters were stored at –20°C and biomolecule filters were stored at -80°C.

Carbon fixation experiments had to be carried out in batch cultures because ^13^C-DIC labeling levels throughout the experiment had to be kept constant, which is difficult to achieve in air-flushed chemostats. Because the remaining culture in the flasks could not be air-bubbled for the same reason, we added additional unlabeled sodium bicarbonate to a final concentration of 2 mM in order to avoid DIC-limitation during the experiment. Thereafter, all culture flasks (volume between 450 and 650 mL) were enriched with ^13^C-sodium bicarbonate (99% ^13^C) to a final labeling concentration of ∼5% of total DIC concentration. Concentrations and absolute ^13^C-DIC enrichment were measured as previously described (Grosse et al., 2015). Culture flasks were closed airtight and incubated for 24 h at a constant rotation (60 rpm), 20°C and 40 μmol photons m^-2^ s^-1^ light intensity, assuring conditions resembling those of the chemostats. After 24 h samples were taken stable isotope analysis of DIC, POC/PON, and biomolecules and stored as described above until analysis.

### Laboratory Analysis and Biomolecule Extractions

Dissolved inorganic nitrogen (DIN = nitrate + nitrite) and DIP (phosphate) concentrations were analyzed using standard colorimetric methods (Grasshoff et al., 1983).

Detailed descriptions of POC/PON analysis, extraction protocols for biomolecules (CH, AA, and FA), EA-, LC-, and GC/C-IRMS systems as well as compound separation protocols and conditions have been published in detail elsewhere (Grosse et al., 2015 and references therein). In short, frozen POC/PON filters were lyophilized overnight, acidified, and subsequently packed into tin cups before the analysis of organic carbon and nitrogen content and δ^13^C values by EA-IRMS. The POP content was quantified with inductive coupled plasma spectroscopy after digestion with 10 mL of 65% HNO_3_ (ICPOES; Perkin Elmer Optima 3300 DV; Nieuwenhuize and Poley-Vos, 1989). CH samples were acid hydrolyzed and analyzed for concentrations and ^13^C-labeling of individual CH by LC/IRMS using an Aminex HPX-87H column, which separates glucose from all other carbohydrates, while galactose, xylose, mannose, and fructose co-elute in a second peak. A third peak contains fucose, arabinose, and ribose. Glucose is also part of storage compounds and was therefore reported separately from all other CH, which are hereafter referred to as structural CH. AA samples were acid hydrolyzed and analyzed by LC/IRMS using a Primsep A column, which separates a total of 17 individual AA (McCullagh et al., 2006). Due to the analytical procedures glutamate and glutamine co-elute as do aspartate and asparagine, formed one peak each. Other detected AA included threonine, valine, methionine, isoleucine, leucine, lysine, histine, phenylalanine, argin, serine, glycine, alanine, proline, cystine, and tyrosine. Because of their very low concentrations, cysteine and methionine were excluded in the data analysis.

Fatty acid samples were extracted following the protocol of Bligh and Dyer (1959) and subsequently separated into storage lipids (triglycerides), glycolipids and phospholipids by silicate column chromatography. However, it has been shown that the phospholipid fraction also contains other non-P containing intact polar lipids (Heinzelmann et al., 2014). The glycolipid- and phospholipid fractions were therefore combined and are further referred to as structural, membrane-derived lipids. After derivatization to fatty acid methyl esters, they were analyzed and the ^13^C measured by GC/C-IRMS using the column BPX-70.

Biosynthesis rates of each individual compound were calculated from ^13^C incorporation rates according to Grosse et al. (2015), and were added up in order to obtain values for each biomolecule group (essential and non-essential AA, storage and structural FA, and storage (glucose) and structural CH) (see Supplementary Materials for details). Throughout this text, biomolecule concentrations and biosynthesis rates were reported relative to cumulative POC concentrations and C-fixation rates (AA + FA + CH = 100%), respectively. Unidentified biomolecules were included only in total POC concentrations and bulk C-fixation rates.

### Statistical Analysis

To explore differences in amino acid composition between different nutrient limitations and communities, principle component analysis (PCA) was performed using AA data. Data for the relative contribution (%) of (*i*) individual AA concentrations to total AA concentrations (nmol C μmol POC^-1^) and (*ii*) individual AA synthesis to total AA synthesis (nmol C μmol POC^-1^ d^-1^) was used. The package CRAN:factoMineR in the open source software R was used for the PCA analysis using a correlation matrix.

## Results

### Resource Limitation

Both the MNHP and LNHP chemostats received media with low DIN:DIP ratios of 1.28 and 0.5, respectively. Their communities decreased the DIN concentrations from 160 μM (MNHP) and 64 μM (LNHP) in the medium to 2 μM in the steady-state chemostat, while the DIP concentrations remained high at 49 and 46 μM, respectively. DIN:DIP ratios in both chemostats were decreased to 0.04, indicating that the communities were limited by N (**Table 1** and **Figure 1**).

The HNMP and HNLP chemostats received media with high DIN:DIP ratios of 200 and 500, respectively. Nutrient uptake by the phytoplankton increased DIN:DIP ratios in the chemostats further to 275 and 2380, respectively (**Table 1**). The DIP concentrations decreased from 10 μM (HNMP) and 4 μM (HNLP) in the medium to 3 and 0.5 μM, respectively, in the steady-state chemostats, while DIN concentrations remained high at 825 and 1190 μM. The high DIN:DIP ratios, as well as the low DIP concentrations, point at P-limitation in these two chemostats (**Figure 1**).

Three chemostats received media with DIN:DIP ratios of 16 (LNLP, MNMP, HNHP) and nutrient uptake by the phytoplankton reduced the DIN:DIP ratios in the chemostats to 1, 2, and 6 for LNLP, MNMP, and HNHP, respectively. Although those ratios might be interpreted as N-limitation, both DIN and DIP concentrations were very low in the LNLP and MNMP chemostats (**Table 1**), and therefore suggested N+P co-limitation (**Figure 1**). In contrast, nutrients in the HNHP chemostat remained high with 181 μM DIN and 29 μM DIP (**Table 1**). At the same time, the high biomass (26.3 mM POC) decreased light levels, inducing light-limitation in this chemostat (**Figure 1**).

**FIGURE 2:**
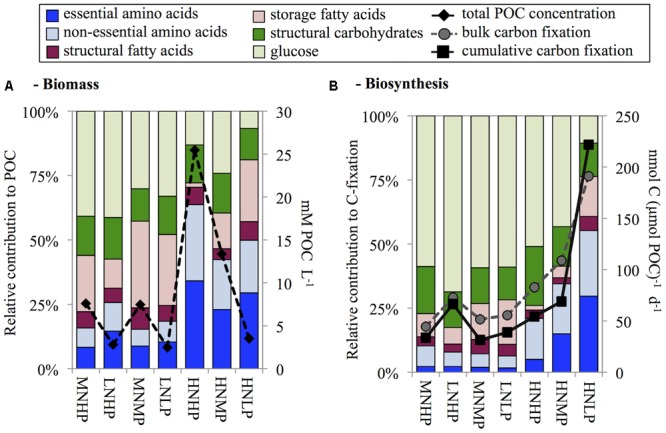
Overview of biomolecule composition of biomass **(A)** and biosynthesis **(B)** sorted by DIN:DIP ratios in the chemostats before the ^13^C addition experiment. Biomass concentrations are added to panel **A** as total particulate organic carbon (POC), and both bulk and cumulative carbon fixation are added to panel **B**.

Phytoplankton biomass ranged from 3.0 to 26.3 mM POC and increased with increasing DIN and DIP concentrations in the mineral medium (**Figure 2A** and **Table 1**). POC:PON ratios in N-limited and N+P co-limited chemostats ranged between 19 and 26 and were lower in light- and P-limited chemostats (**Table 1**). Extremely high POC:POP ratios (>1000) were found in the P-limited chemostats. The MNMP chemostat also showed extremely high POC:POP ratios, consistent with the idea that this community was co-limited by N and P (**Table 1**). PON:POP ratios were >100 in P-limited chemostats and ranged between 18 and 53 in all others.

Based on these POC:PON and POC:POP ratios and total C-fixation rates, we calculated daily DIN and DIP requirements. The N-limited and N+P co-limited chemostats had lowest DIN requirements of only 1.8 nmol DIN μmol POC^-1^ d^-1^ for the MNHM chemostat and slightly higher values for the LNLP, LNHP and MNMP chemostats. The P-limited HNLP required 24.3 nmol DIN μmol POC^-1^ d^-1^. The HNHP and HNMP chemostats required similar amounts of DIN with 9.9 and 9.3 nmol DIN μmol POC^-1^ d^-1^.

Phytoplankton in the P-limited and N+P co-limited chemostats had DIP requirements ranging from 0.04 to 0.14 nmol DIP μmol POC^-1^ d^-1^. The DIP-requirements in the other chemostats ranged from 0.06 to 0.22 nmol DIP μmol POC^-1^ d^-1^ (**Table 1**).

### Community Composition

Differences in DIN and DIP concentrations in media, and resulting DIN:DIP ratios shaped the community structure in all seven chemostats. Based on microscopic observations and flow cytometry (Accuri C6 flow cytometer, BD Biosciences, San Jose, CA, United States) at least five species could be distinguished in the steady-state chemostats, representing three phytoplankton phyla. Green algae were represented by a *Chlorella* sp., while unicellular cyanobacteria (*Synechococcus* spp.) and diatoms (*Nitzschia agnita* and *N. pusilla)* were represented by at least two taxa each. *Chlorella* sp. and the two strains of *Synechococcus* spp. were distinguished by differences in their chlorophyll and phycocyanin fluorescence as well as their cell size, using flow cytometry. The two diatom species were identified microscopically.

Mixed communities developed in all chemostats. Diatoms dominated one of the N-limited chemostats (LNHP), both N+P co-limited chemostats (LNLP, MNMP), and one of the P-limited chemostats (HNLP), where *N. agnita* was more abundant under N-limited conditions and *N. pusilla* under P-limited conditions. Cyanobacteria dominated in the other N-limited chemostat with diatoms being second most abundant (MNHP). Green algae dominated in the other P-limited chemostat (HNMP), while the light-limited chemostat (HNHP) showed a more even co-dominance of green algae and diatoms (**Table 1**).

### Biochemical Composition and Synthesis

AA contribution to biomass was highest in the light limited HNHP chemostat where it contributed 64% of POC concentration, intermediate in the P-limited HNMP and HNLP chemostats contributing 42% and 50% of POC concentration, respectively, and lowest in the remaining four N-(co-)limited chemostats (16–26% of POC concentration, **Figure 2A**). Glucose concentrations showed an opposite trend to that of total AA and contributed between 7 and 42% to POC concentration.

Storage FA contributions varied considerably (1.6–33% of POC concentration), being lowest in the HNHP chemostat and highest in the MNMP chemostat. However, no DIN:DIP ratio dependent increase or decrease was observed. Structural CH and structural FA showed little variation. Structural CH contributed 14 ± 2% to POC concentrations and structural FA contributed 6 ± 1% to POC concentration (averages ± standard deviation, *n* = 7).

With the dilution rate set to 0.2 d^-1^, we would have expected to find biomass specific C-fixation rates to be ∼200 nmol C μmol POC^-1^ d^-1^. However, only the HNLP chemostat showed expected value, while C-fixation rates of all other chemostats were considerably lower (**Figure 2B**). One likely explanation is the contribution of dead material to the biomass, leading to an underestimation of biomass specific C-fixation rates. The 24 h incubation of the C-fixation experiment induced further nutrient depletion, which may have also been a contributing factor to the decreased specific C-fixation rates. Alternatively, nutrient-stressed phytoplankton can exudate photosynthetically fixed carbon as DOC (Myklestad, 2000; Nagata, 2000), a pool that we have not quantified in this study.

The synthesis rates of all investigated biomolecules summed up to between 61% (MNMP) and 91% (LNHP) of bulk C-fixation (**Figure 2B**). This range was similar to field findings (Grosse et al., 2015) and suggested that 9–32% of bulk carbon fixation ends up in biomolecules that were not investigated in this study, such as nucleic acids (DNA and RNA) or pigments. In the HNLP chemostat, we found a value slightly above 100% (116%). This was also in accordance with field findings of P-limited, diatom-dominated stations in the North Sea (Grosse et al., 2015, 2017) and suggests that the de-novo synthesis of nucleic acids and pigments may have been low. Additionally, the diatoms also formed sticky aggregates and the subsequent splitting of the cultures into equal parts was difficult, which could have caused an experimental error.

With the exception of the HNLP chemostat, the majority of fixed C was still in the glucose fraction after 24 h (43–69% of C-fixation). AA synthesis was highest in the HNLP chemostat contributing 55% of C-fixation and decreased to values between 6 and 10% of C-fixation in chemostats with DIN:DIP ratios below ≤2. The HNHP chemostat also showed decreased AA synthesis, accounting for 21% of C-fixation (**Figure 2B**).

**FIGURE 3:**
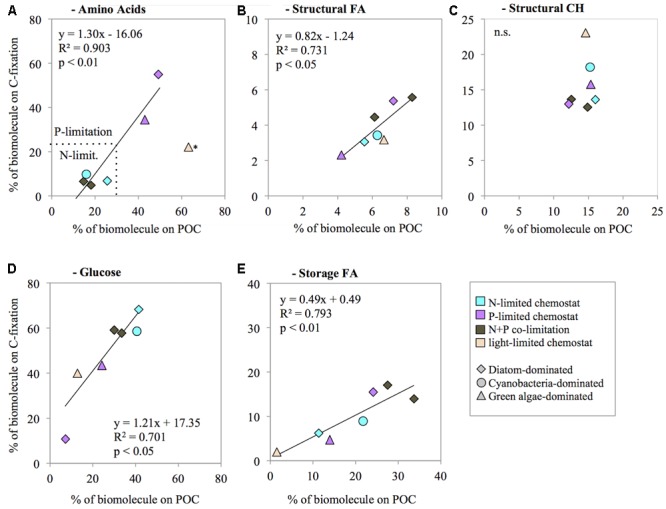
Correlation between the contribution of biomolecules to biomass (% of POC concentration) and their contribution to biosynthesis (% of C-fixation), for amino acids **(A)**, structural fatty acids **(B)**, structural carbohydrates **(C)**, glucose **(D)**, and storage fatty acids **(E)**. The prevailing nutrient limitation is indicated by symbol color, the dominant phytoplankton group is indicated by symbol shape. ^∗^the light-limited chemostat was treated as an outlier in panel **A**.

We investigated whether the contribution of biomolecules to biomass (% of POC concentration) was correlated with their contribution to biosynthesis (% of C-fixation). The contribution of total AA to biomass was not significantly correlated with the contribution of AA to biosynthesis (*R*^2^ = 0.46, *n* = 7, n.s.; **Figure 3A**). The data suggest that the light-limited chemostat (HNHP) was an outlier, however. Possibly the N concentration was depleted during the 24 h incubation for the C-fixation measurements, thereby suppressing AA synthesis. Removal of this outlier resulted in a significant correlation between the AA contribution to biomass and to biosynthesis (*R*^2^ = 0.90, *n* = 6, *p* < 0.01; **Figure 3A**). Structural FA showed a significant correlation between its contribution to biomass and its contribution to biosynthesis (*R*^2^ = 0.73, *n* = 7, *p* < 0.05; **Figure 3B**). By contrast, structural CH did not show a significant correlation (*R*^2^ = 0.07, *n* = 7, n.s.; **Figure 3C**). Instead, the contribution of structural CH to biomass did not show much variation (mean ± SD of 16 ± 4%), indicating that a fixed proportion of the phytoplankton biomass was invested in structural CH irrespective of nutrient availability. Storage CH (glucose) and storage FA both showed a significant correlation between their contribution to biomass and their contribution to biosynthesis (glucose: *R*^2^ = 0.71, *n* = 7, *p* < 0.05, **Figure 3D**; storage FA: *R*^2^ = 0.79, *n* = 7, *p* < 0.01; **Figure 3E**).

### Individual Amino Acids

**FIGURE 4:**
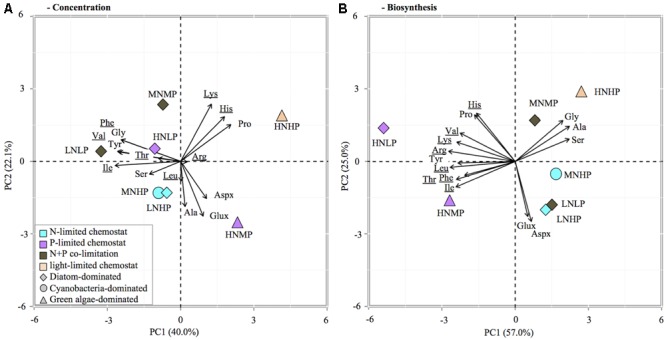
Principle component analysis (PCA) biplot of relative contribution of individual AA to total AA concentration in POC **(A)** and total AA synthesis **(B)**. Symbol color indicates limitation, symbol shape refers to dominant phytoplankton group. The analysis included 15 amino acids: glutamate/glutamine (Glux), aspartate/asparagine (Aspx), threonine (Thr), valine (Val), isoleucine (Ile), leucine (Leu), lysine (Lys), histidine (His), phenylalanine (Phe), argin (Arg), serine (Ser), glycine (Gly), alanine (Ala), proline (Pro), and tyrosine (Tyr).

Principle component analysis of the relative contribution of individual AA to total AA concentration and C-fixation rates revealed differences between phytoplankton groups as well as nutrient limitations (**Figure 4**). PCA analysis of AA concentrations indicated that 62% of the variation was explained by the first two axes. The first axis separates the AA lysine, histine, proline, glutamate/glutamine, aspartate/asparagine, and alanine from all others and caused a separation of chemostats dominated by diatoms and cyanobacteria from chemostats dominated by green algae (HNHP, HNMP, **Figure 4A**), demonstrating a phytoplankton group specific separation of AA. The second axis showed that lysine, histine, and proline were associated with the HNHP chemostats, whereas glutamate/glutamine, aspartate/asparagine and alanine were associated with the HNMP chemostat, demonstrating a nutrient specific effect on AA distribution in chemostats with green algae dominance. A nutrient related separation was less distinct in diatom and cyanobacteria dominated chemostats, however, N-limited chemostats seemed to associate with serine and alanine, while the N+P-co-limited MNMP chemostat drifted towards Lys (**Figure 4A**).

A pronounced nutrient specific separation was visible in the AA biosynthesis data (**Figure 4B**). The first two axes in the PCA of AA biosynthesis explained 82% of the variation within the samples. A separation between nutrient limitations was visible, along the first axis. The P-limited chemostats (HNLP, HNMP) were associated with all essential AA and proline, while all other chemostats were associated with all non-essential AA (except proline). A separation along the second axis occurred as well; chemostats with DIN:DIP ratios of 0.04 and 2 (LNHP, MNHP, LNLP) associated with glutamate/glutamine and aspartate/asparagine and chemostats with DIN:DIP ratios of 1 and 6 (MNMP, HNHP) associated with alanine, serine, and glycine.

## Discussion

### Effects on Phytoplankton Stoichiometry

Although chemostat experiments cannot reproduce the full complexity of marine ecosystems, they provide an excellent tool to study the response of marine phytoplankton to different N and P levels under highly controlled conditions. In our study, four different limitations were encountered among the chemostats (**Figure 1**). Chemostats receiving media with low DIN:DIP ratios became N-limited, whereas chemostats receiving media with high DIN:DIP ratios became P-limited. Chemostats that received DIN:DIP ratios at the optimal Redfield ratio (Redfield et al., 1963) developed a co-limitation by N+P at low and medium DIN and DIP concentrations. The relatively low DIN:DIP ratios in these two chemostats indicate that the N+P co-limitation might be tending somewhat more to N- than to P-limitation. The chemostat that received high DIN and DIP concentrations developed a high biomass that induced light-limitation through self-shading (Brauer et al., 2012).

Phytoplankton PON:POP ratios and POC:POP ratios in the HNMP and HNLP chemostats were very high, supporting the conclusion that these cultures were P-limited. Interestingly, though, storage CH (glucose) and storage FA contents of the phytoplankton were lower in these P-limited chemostats (**Figure 2A**) than in the N+P co-limited chemostat MNMP, which showed similar POC:POP ratios. This may indicate that the high POC:POP ratios under solely P-limited conditions were mainly due to low cellular P contents, while the high POC:POP ratio in the MNMP chemostat was additionally determined by a higher accumulation of C-rich storage compounds. Overall, P-deficient phytoplankton tend to have a low nutritional value for a variety of herbivorous zooplankton (Plath and Boersma, 2001), which can negatively affect growth rates of zooplankton and larvae of fish and shellfish (Malzahn and Boersma, 2012; Schoo et al., 2014) and consequently induces changes in the entire food web (Sterner et al., 1993).

Conversely, PON:POP ratios were lowest in the MNHP and LNHP chemostats but do not point at a severe N limitation, since PON:POP ratios of 18 to 30 can also be found under nutrient replete conditions or in communities transitioning from N to P limitation (Geider and LaRoche, 2002). The depletion of DIN did, however, cause an accumulation of C-rich storage CH (glucose) and storage FA, and increased the POC:PON ratio to values typical for N-limited phytoplankton (Geider and LaRoche, 2002).

### Biomolecule Dynamics under Different Resource Limitations

It should be noted that no nutrients were added during the ^13^C-incubations, which will have resulted in a decrease of available nutrients (compared to chemostat conditions) and may have affected the outcome of these carbon fixation experiments to some extent. While the biomolecule concentration of cultures was determined by the conditions in the chemostats (long-term adaptation), the biomolecule synthesis rates will have been affected immediately by decreasing nutrient availabilities during the ^13^C-incubations. N-, P-, and N+P co-limited cultures probably became exhausted of DIN and/or DIP. The light limited HNHP culture may have also exhausted the DIN concentration and probably became co-limited by N and light during the incubation experiments. This was most evident in AA biosynthesis rates, which were much lower than expected (see the outlier in **Figure 3A**). AA will be mainly used in protein synthesis, and nitrogen limitation causes changes in the levels of transcription and translation (Yang et al., 2011; Alipanah et al., 2015). Several studies have demonstrated that gene expression, especially of the photosystem and ribosomal genes, starts to change within a few hours after removal or addition of nitrogen (Morey et al., 2011; Krasikov et al., 2012), indicating that AA synthesis may indeed decline rapidly in response to a decrease in N availability.

AA contents and synthesis rates were closely linked to N availability. In particular, in N-limited and N+P co-limited phytoplankton AA contributions to POC and C-fixation were very low, whereas AA contents and synthesis rates were much higher under light-limited and P-limited conditions (**Figure 2**). The reduction of AA synthesis under N-limited conditions appears at odds with model predictions of Klausmeier et al. (2004), where *both* N-limited and P-limited phytoplankton invest in nutrient uptake proteins and hence have relatively high N:P ratios. Instead, our findings are more in agreement with the model of Loladze and Elser (2011), which predicts that N limitation slows down AA synthesis and thereby lowers organismal N:P ratios, whereas P limitation does not constrain AA synthesis and results in high cellular N:P ratios.

The correlation trendline between AA contribution to POC concentrations and AA contribution to C-fixation crosses the x-axis at a value of ∼12% (**Figure 3A**), which can be interpreted as the minimum AA concentration necessary in the POC under N-starvation. In other words, this is the minimum amount of AA needed to maintain cell functions under zero growth. We found slightly higher values in the North Sea, where required minimum concentrations of AA in POC were ∼17% (Grosse et al., 2017). The small difference between our laboratory results and these field observations may have been caused by the contribution of micro- and mesozooplankton and debris in the field, which can also be sources of AA.

Accumulation of storage CH (glucose) showed a pattern opposite to AA synthesis. CH contents were lowest under light-limited and P-limited conditions whereas high levels of storage CH accumulated in N-limited phytoplankton. A similar contrast between AA synthesis and CH accumulation was obtained in short-term experiments with natural phytoplankton during a series of research cruises on the North Sea, where N addition increased AA synthesis of N-limited phytoplankton within 24 h while CH storage decreased concomitantly (Grosse et al., 2017).

A direct relationship between P availability and rRNA synthesis has been established previously (Hessen et al., 2004; Van Mooy and Devol, 2008) and with evidence of P-limitation becoming more prevalent in coastal seas (Sylvan et al., 2007; Xu et al., 2008; Burson et al., 2016) measurements of nucleic acids concentrations and biosynthesis should be included into future studies of biomolecule dynamics. A method to detect ^13^C incorporation into DNA and RNA nucleotides recently became available (Moerdijk-Poortvliet et al., 2014). Biomass requirements for this method, however, are much higher than for the biomolecules investigated here and the culture volumes in our chemostats did not allow for the additional sampling of this parameter.

### Effects on Amino Acid Composition

The different nutrient treatments in our experiments had considerable effects not only on the AA content, but also on the AA composition of marine phytoplankton. Similar results were found in a recent field study in the North Sea, where N-limited communities were associated especially with glutamate/glutamine and aspartate/asparagine, while P-limited communities were characterized by higher contributions of essential AA (Grosse, 2016). Together, these findings provide an interesting new perspective on individual AA dynamics in the water column. Previous studies on the geochemical composition of particulate organic matter assumed that the AA composition of marine phytoplankton is more or less constant (e.g., Dauwe et al., 1999). In contrast, our lab experiments and recent field study (Grosse, 2016) point at consistent variation in the AA composition of marine phytoplankton depending on the growth conditions.

Proline showed high contributions to the POC concentration in the light limited chemostat (HNHP, Supplementary Table S1). Under N-replete conditions, proline can be used as an osmoprotectant, which is replaced by compounds such as dimethylsulfoniopropionate under N-depleted conditions (Bromke et al., 2013), and only the HNHP chemostat had sufficient DIN available to suggest proline may have been important for osmoregulation. Glutamate/glutamine and aspartate/asparagine are directly synthesized from glycolysis and TCA intermediates, and thereby, constitute precursors for the synthesis of AA with longer synthesis pathways (especially essential AA). They were therefore first affected by changing N availabilities. The other non-essential AA (serine, alanine, and glycine) had higher contribution to C-fixation than they had in biomass (over-synthesis), indicating the transformation into AA “down the line” was not completed after 24 h, which was confirmed by all essential AA showing lower synthesis compared to their percentage contribution in biomass (under-synthesis). The results also showed that the degree of “over-” or “under-synthesis” was greater under N-limitation than under P-limitation, suggesting AA synthesis from non-essential to essential AA occurred slower under N-limitation. For example, the non-essential alanine contributed 12% to total AA synthesis in the two P-limited chemostats, whereas the synthesis contribution increased in N-limited phytoplankton to between 17 and 29% (Supplementary Table S1). Similarly, large differences were found in glutamate/glutamine, serine, glycine, and leucine, while differences in other AA were observed but at a much lower scale. N and P-limitation affected biosynthesis of total AA and in addition turnover times of precursor may be longer because the conversion of non-essential to essential AA relies on numerous additional enzymes, proteins themselves, and their production may be reduced under N-limitation as well.

The distribution of individual FA was also analyzed but we did not find any nutrient-dependent relationships. These were most likely concealed by pronounced differences in FA composition between different phytoplankton groups (Dijkman and Kromkamp, 2006).

## Conclusion

The chemostat experiments showed that changes in N and P supply lead to substantial changes in the biochemical composition as well as species composition of phytoplankton communities. Although natural phytoplankton communities are clearly more complex than laboratory chemostats, the nutrient-dependent shifts in biomolecule composition and biosynthesis from these simplified chemostat experiments are generally in agreement with results from natural phytoplankton communities in the North Sea (Grosse, 2016; Grosse et al., 2017). In particular, our experimental results show that shifts from N limitation to P limitation, as observed in coastal waters like the North Sea, will not only increase the N:P and C:P stoichiometry of phytoplankton but will also increase their total amino acid content, alter their amino acid composition and reduce their cellular carbohydrate storage.

Future studies may build on this work, by expanding beyond the elemental stoichiometry of phytoplankton to further elucidate the range of adaptations in biochemical composition of different phytoplankton species, and their implications for, e.g., phytoplankton growth rates, DOM production, microbial loop activity, the production of secondary metabolites and the nutritional quality of phytoplankton as food for higher trophic levels.

## Author Contributions

JG and AB designed research; MS, JH, and HB supervised design and implementation, JG and AB performed research and data analysis; JG, AB, MS, JH, and HB interpreted the data and wrote the paper.

## Conflict of Interest Statement

The authors declare that the research was conducted in the absence of any commercial or financial relationships that could be construed as a potential conflict of interest.
