# Supplementary material for: From Ecological Stoichiometry to Biochemical Composition: Variation in N and P Supply Alters Key Biosynthetic Rates in Marine Phytoplankton
Source: Front Microbiol. 2017 Jul 12;8:1299. doi: 10.3389/fmicb.2017.01299 (PMC5506227; doi:10.3389/fmicb.2017.01299)
Supplement: Supplementary file 1 [file Table_1.PDF]

Supplementary Material

From Ecological Stoichiometry to Biochemical Composition: Variation in N and P Supply Alters Key Biosynthetic Rates in Marine Phytoplankton

Julia Grosse*, Amanda Burson, Maayke Stomp, Jef Huisman, Henricus T. S. Boschker

*** Correspondence:** Julia Grosse: jgrosse@geomar.de

1 Material & Methods

Calculation of biosynthesis rates

Biosynthesis rates of the different molecules were calculated from ^{13}C incorporation according to Grosse et al. (2015). In short, carbon stable isotope ratios are expressed in the $\delta^{13}C$ notation:

$\delta^{13}C_{sample}$ (‰) = ((R_{sample}/R_{VPDB}) - 1) x 1000,

where R_{sample} and R_{VPDB} denote the $^{13}C/^{12}C$ ratio in the sample and the international standard, Vienna Pee Dee Belemnite (for carbon $R_{VPDB} = 0.0111802 \pm 0.0000009$), respectively.

Incorporation of ^{13}C into bulk carbon as well as individual compounds is reflected as excess (above background) ^{13}C in equation 1:

Excess $^{13}C_{sample}$

$$= \left[\left(\frac{(\delta^{13}C_{sample}/1000+1)\times R_{VPDB}}{(\delta^{13}C_{sample}/1000+1)\times R_{VPDB}+1}\right)-\left(\frac{(\delta^{13}C_{background}/1000+1)\times R_{VPDB}}{(\delta^{13}C_{background}/1000+1)\times R_{VPDB}+1}\right)\right]\times \text{concentration}_{sample} \quad (1),$$

where $\delta^{13}C_{sample}$ refers to the $\delta^{13}C$ value of bulk material (POC) or the compound of interest at the end of the incubation, $\delta^{13}C_{background}$ denotes the $\delta^{13}C$ value of the unlabeled POC or compounds before the addition of ^{13}C-DIC, $\text{concentration}_{sample}$ denotes the concentration of POC or compound in nmol of carbon per liter (nmol C L^{-1}) at the end of the incubation.

Similarly, the enrichment of the DIC pool with ^{13}C has to be calculated (Equation 2) in order to determine total carbon incorporation.

$$\text{Enrichment DIC} = \left(\frac{(\delta^{13}C_{DICsample}/1000+1)\times R_{VPDB}}{(\delta^{13}C_{DICsample}/1000+1)\times R_{VPDB}+1}\right)-\left(\frac{(\delta^{13}C_{DICbackground}/1000+1)\times R_{VPDB}}{(\delta^{13}C_{DICbackground}/1000+1)\times R_{VPDB}+1}\right) \quad (2),$$

where $\delta^{13}C_{DICsample}$ refers $\delta^{13}C$ of DIC in culture flasks at the end of the incubation and $\delta^{13}C_{DICbackground}$ denotes $\delta^{13}C$ of DIC before the addition of ^{13}C-DIC.

Biosynthesis rates (nmol C (µmol POC)$^{-1}$ d^{-1}) are calculated as followed:

$$\text{Biosynthesis rate} = \left[\left(\frac{\text{Excess }^{13}C_{sample}}{\text{Enrichment DIC}}\right)\Big/ POC_{concentration}\Big/ \Delta t\right]\times 24 \quad (3)$$

where $POC_{concentration}$ is the concentration of POC (µmol L^{-1}) at the end of the incubation and Δt is the incubation time in hours. A multiplication with 24 results in daily rates. A normalization of rates to biomass allows comparison between different phytoplankton communities and chemostats.

Concentrations and biosynthesis rates were calculated for each individual compound. Concentrations and biosynthesis rates of subgroups (e.g. essential/non-essential AA, storage/structural CH/FA) or total macromolecule groups (total fatty acids, amino acids and carbohydrates) were obtained by summing all individual biosynthesis rates within that group.

2 Supplementary Table

Supplementary Table 1: Contributions of individual amino acids to total amino acid concentration (% AA conc.) and total amino acid synthesis (% AA synth.) in each chemostat, separated by non-essential and essential amino acids [aspartate/ asparagine (Aspx), glutamate/ glutamine (Glux), alanine (Ala), serine (Ser), glycine (Gly), tyrosine (Tyr), proline (Pro), phenylalanine (Phe), lysine (Lys), threonine (Thr), isoleucine (Ile), leucine (Leu), valine (Val), histidine (His), argin (Arg)].

			MNHP	LNHP	MNMP	LNLP	HNHP	HNMP	HNLP
**non-essential amino acids**	Aspx	% AA conc.	5.28	3.76	4.19	3.64	4.04	5.85	2.68
		% AA synth.	5.05	7.10	3.72	6.99	4.19	7.37	2.88
	Glux	% AA conc.	9.63	8.16	6.76	8.55	8.25	12.39	8.15
		% AA synth.	25.62	19.30	13.35	19.70	6.07	19.36	9.66
	Ala	% AA conc.	9.24	9.84	8.96	9.49	9.35	9.63	9.35
		% AA synth.	17.26	17.28	22.44	21.06	29.05	11.63	12.12
	Ser	% AA conc.	7.78	7.11	6.38	5.25	4.86	5.01	6.71
		% AA synth.	14.86	14.57	14.00	9.17	15.37	6.45	7.68
	Gly	% AA conc.	5.64	5.93	6.10	6.96	5.31	5.15	6.00
		% AA synth.	8.89	7.52	12.96	11.66	16.13	5.81	6.57
	Tyr	% AA conc.	6.10	5.45	6.43	6.77	4.59	5.35	6.47
		% AA synth.	4.40	4.12	4.87	4.34	3.13	4.62	6.02
	Pro	% AA conc.	4.47	4.52	5.61	4.44	11.54	4.57	4.50
		% AA synth.	2.01	1.59	2.19	0.86	2.23	1.93	3.07
**essential amino acids**	Phe	% AA conc.	9.58	9.00	9.57	10.19	8.68	8.51	9.91
		% AA synth.	3.75	7.08	2.86	5.36	5.26	7.23	9.09
	Lys	% AA conc.	3.99	4.11	5.32	4.42	5.65	4.11	4.27
		% AA synth.	1.88	0.79	1.98	0.66	0.87	2.15	3.32
	Thr	% AA conc.	4.64	4.84	5.61	4.89	4.22	5.03	4.39
		% AA synth.	2.42	3.28	3.56	3.04	2.01	4.72	4.39
	Ile	% AA conc.	6.48	6.77	6.40	7.55	5.22	5.72	6.50
		% AA synth.	1.91	3.31	1.88	3.89	1.16	4.59	5.86
	Leu	% AA conc.	16.48	18.37	15.81	15.34	16.20	15.84	16.82
		% AA synth.	5.98	9.00	6.21	6.69	6.59	14.66	16.79
	Val	% AA conc.	8.28	8.46	8.45	9.00	7.55	7.58	8.13
		% AA synth.	5.44	4.71	6.57	6.21	6.18	6.90	8.25
	His	% AA conc.	0.84	0.92	1.06	0.80	1.12	0.90	0.96
		% AA synth.	0.29	0.10	0.65	0.00	0.79	0.74	0.92
	Arg	% AA conc.	1.57	2.77	3.13	2.72	2.71	4.08	4.85
		% AA synth.	0.24	0.26	0.50	0.38	0.26	1.56	3.03
